# Book Reviews

**Published:** 1891-05

**Authors:** 


J. B. Lipincott Company will, beginning with April, issue quarterly thereafter, a work entitled "International Clinics." This work will comprise the best and most practical clinical lectures on medicine, surgery, gynæcology, pediatrics, dermatology, laryngology, opthalmology, and otology, delivered in the leading medical colleges in this country, Great Britain and Canada. These lectures have been reported by competent medical stenographers and thoroughly revised by the professors and lecturers themselves. The object of the work is to furnish the busy practitioner and medical student with the best and most practical clinical instruction in concise form· Each volume will consist of over 350 octavo pages, illustrated with photographic reproductions of important cases.

---

EARACHE.—

℞ Camphorated chloral, 5 parts.
Glycerine, 30 parts.
Ol. sweet almonds, 10 parts.

M. Sig: Saturate cotton and place in the ear; also rub some behind the ear. *Ex.*

